# Anterior Ischemic Optic Neuropathy as a Paraneoplastic Manifestation of Colorectal Cancer

**DOI:** 10.7759/cureus.100070

**Published:** 2025-12-25

**Authors:** Guilherme Ferreira Sacramento, Sérgio Brito, Teresa Mesquita

**Affiliations:** 1 Department of Medicine, Unidade Local de Saúde de Lisboa Ocidental, Lisbon, PRT; 2 Stroke Unit, Hospital Professor Doutor Fernando Fonseca, Amadora, PRT

**Keywords:** colorectal cancer, inflammation, ischemic optic neuropathy, non-arteritic anterior ischemic optic neuropathy (naion), ocular ischemia

## Abstract

Non-arteritic anterior ischemic optic neuropathy (NAION) is one of the most common causes of acute optic nerve-related vision loss. Its non-arteritic form is strongly associated with vascular risk factors. The role of systemic inflammation in its pathophysiology has increasingly been recognized, and it may coexist with prothrombotic or neoplastic states. We present the case of a patient with sudden vision loss and a diagnosis of NAION in the context of unexplained systemic inflammation, during the workup of which an underlying colorectal carcinoma was identified. This case highlights the importance of a comprehensive clinical approach to ischemic events, including the investigation of underlying inflammatory and neoplastic causes.

## Introduction

Anterior ischemic optic neuropathy (AION) results from hypoperfusion of the optic disc, causing sudden, painless visual loss in one eye, often with altitudinal visual field defects. It is a leading cause of optic nerve-related blindness, with its pathogenesis, clinical features, and management remaining subjects of discussion [1]. AION may present as an arteritic form, typically linked to giant cell arteritis, or as non-arteritic AION (NAION), which is more common and associated with classic vascular risk factors [2]. Prognosis is variable, with some spontaneous recovery, but permanent deficits are common. Diagnosis is clinical and primarily aimed at excluding arteritic AION. Corticosteroids have no proven benefit in non-arteritic cases and are used mainly to rule out arteritic causes.

Emerging evidence suggests that systemic inflammation and prothrombotic states may increase susceptibility to optic nerve ischemia [3,4]. Although NAION is most commonly attributed to vascular risk factors such as hypertension, diabetes, or optic disc crowding, a small number of case reports have described NAION in patients with systemic malignancies, particularly hematologic cancers and, more rarely, solid tumors [5,6]. Paraneoplastic manifestations in neuro-ophthalmology refer to visual or neurologic deficits caused indirectly by systemic malignancies, often via immune-mediated or prothrombotic mechanisms [4]. To our knowledge, there is currently no established evidence linking NAION and colorectal cancer. A literature review identified only one case report describing NAION in a patient with colorectal cancer receiving chemotherapy. A multifactorial pathogenesis involving preexisting anatomical risk factors has been proposed [7]. Although systemic malignancy is rarely considered in the differential diagnosis of NAION, its recognition may be important in atypical presentations, particularly when accompanied by systemic inflammatory features or other warning signs. We present a case of NAION in a patient with systemic inflammatory syndrome who was subsequently diagnosed with colorectal cancer, exploring potential pathophysiologic links.

## Case presentation

A 67-year-old man with type 2 diabetes, hypertension, peripheral arterial disease, and bilateral carotid stenosis presented with acute, painless vision loss in the left eye. Two weeks earlier, he had experienced sudden right-sided hearing loss, confirmed by tympanometry and audiometry. Magnetic resonance imaging revealed no underlying etiology, and he remained under otorhinolaryngology follow-up, with an idiopathic cause presumed. There were no neurologic deficits, headache, jaw claudication, or fever. Unintentional weight loss of approximately 10 kg over four months (>5% of body weight) was reported.

Ophthalmologic evaluation revealed decreased visual acuity and sectoral optic disc edema in the inferior temporal arcade. NAION versus branch retinal artery occlusion was considered. Cranial CT and MRI showed no mass lesions or acute ischemia. Bilateral carotid stenosis was confirmed but was hemodynamically insignificant.

Laboratory studies demonstrated anemia of chronic disease and markedly elevated inflammatory markers (erythrocyte sedimentation rate (ESR) >140 mm/h, CRP 11 mg/dL) without evidence of infection. Autoimmune testing was negative (Table 1). Given the suspicion of giant cell arteritis, corticosteroid therapy was initiated. However, a temporal artery ultrasound performed by an experienced operator with specific expertise in giant cell arteritis ruled out the diagnosis. Corticosteroids were subsequently tapered gradually with the goal of complete discontinuation.

**Table 1:** Laboratory studies from the patient ANCA, antineutrophil cytoplasmic antibodies; ANA, antinuclear antibodies; ESR, erythrocyte sedimentation rate; RF, rheumatoid factor

Laboratory studies	Value	Reference range
Hemoglobin (g/dL)	7.8	13.5-17.5
Ferritin (ng/mL)	522	20-250
Iron (µg/dL)	16	33-193
Total iron binding capacity (µg/dL)	223	240-450
Transferrin (mg/dL)	179	200-360
Transferrin saturation (%)	16	20-50
Folic acid (ng/mL)	13.5	2.7-17
Vitamin B12 (pg/mL)	594	160-950
Creatinine (mg/dL)	1.55	0.6-1.2
ESR (mm/h)	>140	<20
CRP (mg/dL)	11	<1
ANA	Negative	-
ANCA	Negative	-
Anti-SSA	Negative	-
Anti-SSB	Negative	-
Anti-dsDNA	Negative	-
RF	Negative	-

**Figure 1:**
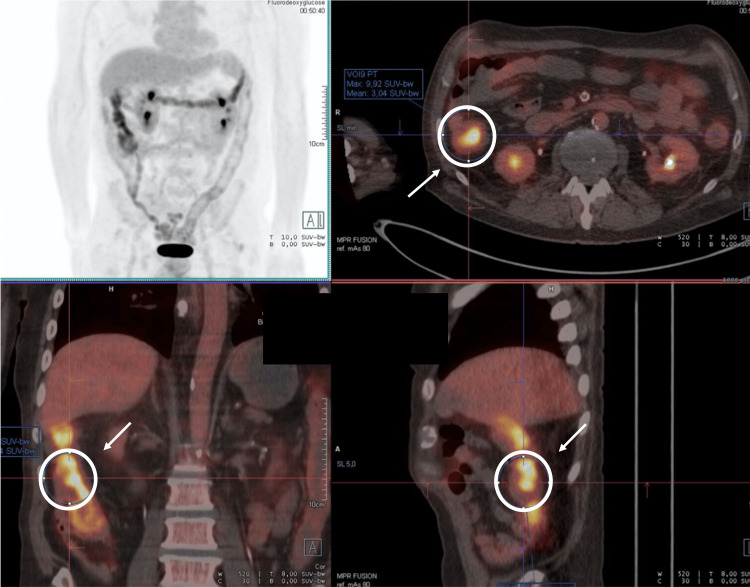
PET scan showing increased FDG uptake in the ascending colon, with no other findings explaining the elevated inflammatory markers

**Figure 2:**
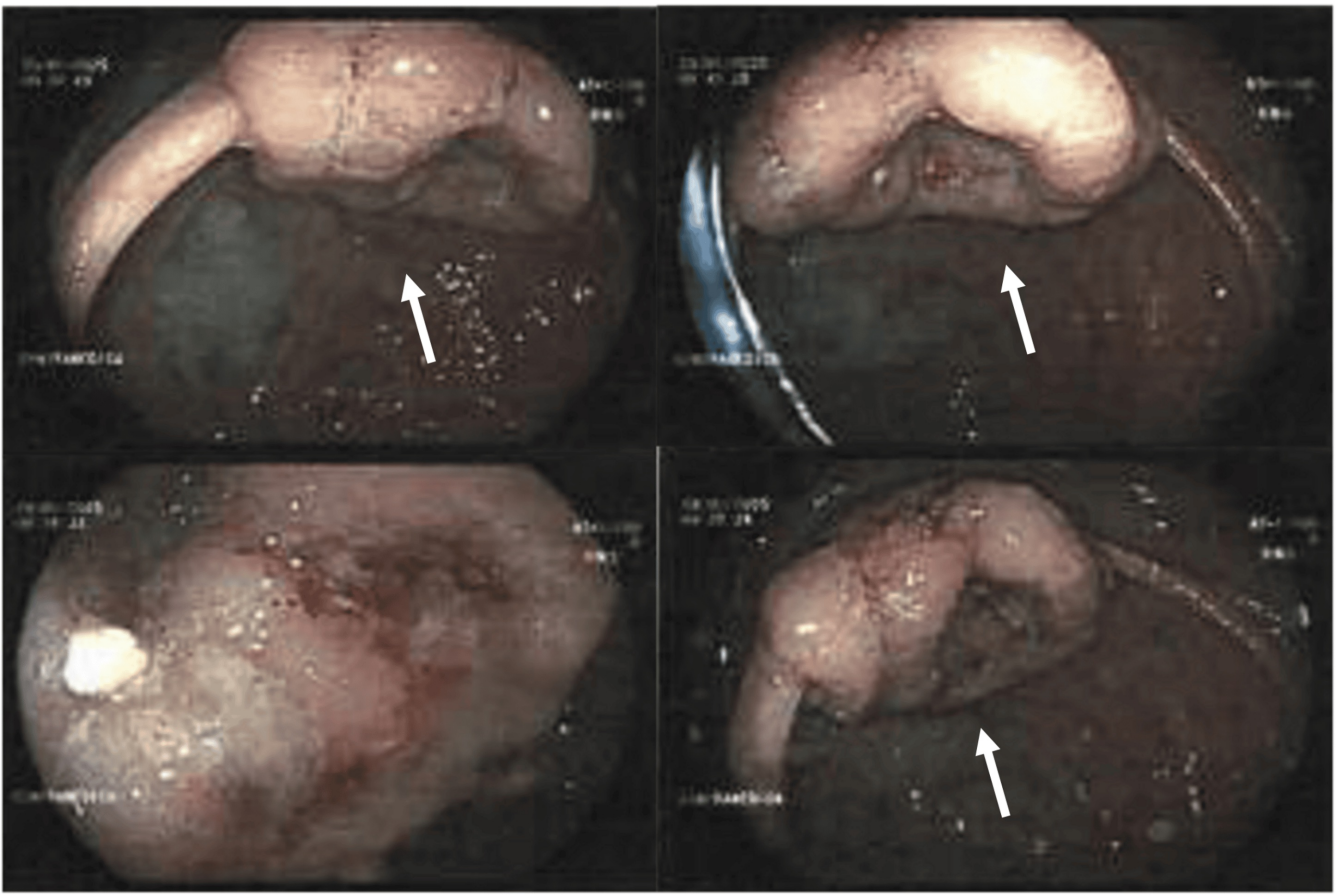
Colonoscopy demonstrating a vegetating colonic lesion Biopsy confirmed colorectal adenocarcinoma.

Persistent systemic inflammation prompted further imaging. PET demonstrated diffuse 18F-FDG uptake in the colon (Figure 1), most pronounced in the ascending colon, with no evidence of vasculitis. Colonoscopy revealed a vegetating lesion, and biopsy confirmed colorectal adenocarcinoma (Figure 2).

A staging workup revealed a colorectal adenocarcinoma classified as T1N0M0 according to the TNM system. Laboratory evaluation showed a carcinoembryonic antigen level of 5.12 ng/mL, CA19-9 of 42 U/mL, and lactate dehydrogenase of 224 U/L.

The patient underwent surgical management one month later, with no adjuvant therapy indicated. While still undergoing corticosteroid tapering, ophthalmologic evaluation documented some improvement in visual acuity prior to surgical management of the underlying malignancy. Visual recovery remained incomplete in the early postoperative period; however, over the subsequent months, the patient demonstrated gradual further improvement in visual function.

The patient’s inflammatory markers also improved over time. After initiation of corticosteroid therapy, the ESR decreased but remained elevated at 63 mm/h. Following hemicolectomy, when the patient was no longer receiving corticosteroids, ESR further declined to 12 mm/h. CRP similarly decreased and remained consistently low thereafter.

At one-year follow-up, the patient remains disease-free.

## Discussion

NAION results from hypoperfusion of the short posterior ciliary arteries, typically in the setting of atherosclerotic risk factors such as diabetes, hypertension, or sleep apnea [8,9]. Although historically considered a purely microvascular condition, systemic inflammatory states are now recognized as potential modulators of optic nerve ischemia risk through mechanisms involving endothelial dysfunction, increased plasma viscosity, and coagulation activation. This creates a pathophysiological continuum linking vascular, prothrombotic, and inflammatory factors, which may amplify optic disc vulnerability [10,11].

In this case, careful exclusion of giant cell arteritis, supported by a negative temporal artery ultrasound and absence of typical clinical features, prompted reevaluation of the significance of the markedly elevated inflammatory markers. Persistently high ESR without evidence of infection or autoimmune disease led to further etiologic investigation, ultimately revealing colorectal adenocarcinoma. This finding is noteworthy, as NAION has not previously been described as an initial manifestation of colorectal malignancy.

Although extremely rare, published case reports describe optic neuropathy associated with colorectal cancer. Examples include paraneoplastic optic neuropathy directly linked to colon adenocarcinoma [12], AION occurring in patients receiving chemotherapy for colorectal cancer (e.g., FOLFOX) [7], and broader paraneoplastic neuro-ophthalmic syndromes in which solid tumors, including colorectal cancer, may induce optic nerve dysfunction via immune-mediated, thrombotic, or infiltrative pathways [13].

The association between malignancy and ocular ischemic events may occur through several mechanisms [3,14]. First, cytokines such as IL-6, TNF-α, and IL-1β, which are typically elevated in malignancy, promote endothelial dysfunction, upregulate adhesion molecules, and activate platelets. These processes facilitate microthrombosis, particularly in terminal circulatory territories such as the optic disc. Second, many solid tumors can induce paraneoplastic syndromes mediated by autoimmune or prothrombotic mechanisms, which have been described in various neuro-ophthalmologic disorders [4].

Existing literature provides isolated instances supporting a possible temporal and mechanistic association between colorectal malignancy and optic neuropathy, although causality remains unproven, and alternative explanations, such as treatment-related effects or traditional vascular risk factors, cannot be excluded.

Although no specific autoimmunity was identified in this patient, an immune- or thrombotic-mediated mechanism may have contributed to his optic ischemic event. Moreover, persistent systemic inflammation may have lowered the optic nerve perfusion threshold in a patient with multiple cardiovascular risk factors. Thus, inflammation related to the active malignancy diagnosed during hospitalization could plausibly have acted as a precipitating factor.

The temporal relationship also deserves emphasis: NAION preceded the diagnosis of colorectal cancer, potentially representing an early marker of underlying systemic disease. Although causality cannot be proven, this case underscores the importance of considering malignancy in patients with persistently elevated inflammatory markers of unknown origin, particularly when unexpected vascular phenomena coexist.

Another noteworthy element is the sudden sensorineural hearing loss occurring weeks before the NAION episode. Although no cases describing the coexistence of these two conditions have been identified in the literature, both share vulnerability to mechanisms of microangiopathy and endothelial dysfunction [15]. Pro-inflammatory or prothrombotic states, such as those associated with malignancy, may precipitate ischemic events in the anterior labyrinth and cochlea. Although a direct causal relationship between sudden hearing loss and NAION cannot be established, the temporal proximity of these events strengthens the hypothesis that both may represent manifestations of a single underlying systemic process, possibly related to a paraneoplastic inflammatory state linked to colorectal adenocarcinoma.

Finally, visual prognosis in NAION remains limited, and treatment is largely supportive, focusing on optimization of vascular risk factors. However, in this patient, early identification of malignancy allowed timely oncologic intervention, potentially preserving quality of life.

## Conclusions

This case illustrates that NAION, a condition most commonly attributed to traditional vascular risk factors, may occasionally occur in patients with systemic malignancy, although current evidence supports only a limited association. While a small number of case reports have described NAION in the context of systemic cancers, predominantly hematologic and, more rarely, solid tumors, no large-scale studies have demonstrated a causal relationship, and NAION is not established as a recognized manifestation of colorectal cancer. In the present case, a direct causal link between colorectal adenocarcinoma and the NAION episode cannot be definitively established. Nevertheless, the absence of alternative explanatory factors, together with the presence of marked, otherwise unexplained systemic inflammation, renders this association noteworthy and justifies its description. This observation aligns with prior reports in which proposed mechanisms, such as paraneoplastic phenomena or hypercoagulable states, have been hypothesized, albeit without consistent supporting evidence.

These findings underscore the importance of a comprehensive, multidisciplinary diagnostic approach in patients presenting with ischemic optic neuropathy, particularly when accompanied by persistently elevated inflammatory markers of unknown origin. In such contexts, consideration of occult malignancy may be warranted, as its recognition has potential implications for patient management and prognosis. Finally, this case reinforces the need for clinicians to integrate vascular, inflammatory, and oncologic perspectives when evaluating atypical ischemic ophthalmologic and, possibly, audiologic presentations, while acknowledging current limitations of the evidence and the absence of a proven pathogenic link.
